# Intraocular pressure and choroidal thickness postural changes in multiple system atrophy and Parkinson’s disease

**DOI:** 10.1038/s41598-021-88250-3

**Published:** 2021-04-26

**Authors:** Maddalena De Bernardo, Giulio Salerno, Marco Gioia, Luigi Capasso, Maria Claudia Russillo, Marina Picillo, Roberto Erro, Marianna Amboni, Paolo Barone, Nicola Rosa, Maria Teresa Pellecchia

**Affiliations:** 1 Department of Medicine, Surgery, and Dentistry “Scuola Medica Salernitana”, University of Salerno, via S. Allende – 84081 - Baronissi, Salerno, Italy; 2 Corneal Transplant Unit, ASL Napoli 1, Naples, Italy

**Keywords:** Parkinson's disease, Diagnostic markers

## Abstract

To evaluate intraocular pressure (IOP) and choroidal thickness (ChT) postural changes in multiple system atrophy (MSA), Parkinson’s disease (PD) patients and healthy controls (HC). 20 MSA patients, 21 PD patients and 14 HC, were examined. All subjects underwent a complete examination, including corneal thickness, ChT, IOP and axial length (AL) measurements. IOP measurement was performed in supine, sitting, and standing positions, whereas ChT in sitting and standing positions. Supine to standing IOP variations were significantly higher in MSA vs PD(p = 0.01) and in MSA vs HC (p < 0.0001), whereas no significant differences were observed between PD and HC (p = 0.397). Mean sub-foveal ChT in MSA was 240 ± 92 μm in sitting position, and 215 ± 94 μm in standing position with a significant reduction (p = 0.008). Mean sub-foveal ChT in PD was 258 ± 79 μm in sitting position, and 259 ± 76 μm in standing position (p = 0.887). In HC it was 244 ± 36 μm in sitting position, and 256 ± 37 μm in standing position with a significant increase (p = 0.007). The significant IOP and ChT postural changes can be considered additional hallmarks of autonomic dysfunction in MSA and further studies are needed to consider them as biomarkers in the differential diagnosis with PD.

## Introduction

Multiple system atrophy (MSA) is a late-onset, sporadic neurodegenerative disease that manifests as an autonomic failure with the variable presence of poorly levodopa-responsive parkinsonism and/or cerebellar ataxia, linked to striatonigral degeneration and olivopontocerebellar atrophy, respectively^1,2^. Non-motor symptoms are strongly associated with neurodegeneration in the brainstem and spinal cord. MSA pathology typically affects multiple regions of the brainstem, including the locus coeruleus, catecholaminergic neurons of the ventrolateral medulla, as well as the dorsal vagal nucleus and the ventrolateral nucleus ambiguous^3–10^. Spinal cord pathology is characterized by neuronal loss in the intermediolateral columns and the Onuf’s nucleus in the lumbosacral region^11,12^. Orthostatic hypotension is a typical dysautonomic feature in MSA and is present in about 60% of patients^2,13^.

Intraocular pressure (IOP) postural variations have been reported in MSA and pure autonomic failure (PAF)^14,15^, and are supposed to be related to autonomic dysfunction at the ocular level. However, the pathophysiology of this finding is not clearly understood. Besides IOP, choroidal thickness (ChT) too could be influenced by postural changes, but, to the best of our knowledge, postural changes in ChT have never been studied in MSA.

Therefore, the aims of our study were:

1. to confirm the IOP postural changes in MSA patients compared with patients affected by Parkinson’s disease (PD) and healthy controls (HC).
2. to assess postural variations in ChT, to better understand the pathophysiology of ocular autonomic dysfunction in MSA.

## Methods

Twenty patients with probable MSA diagnosed according to the current criteria^16^, 21 PD patients fulfilling the current diagnostic criteria^17^, and 14 controls subjects without known systemic or ocular disease were included in the study. Disease duration was recorded, and disease severity was assessed by theUnified Multiple System Atrophy Rating Scale (UMSARS) part II in MSA, and Unified Parkinson's Disease Rating Scale (UPDRS) part III in PD (Table 1). Orthostatic symptoms in MSA patients were graded according to Item 9 of UMSARS I. Presence of orthostatic hypotension, diagnosed according to the current criteria^18^, was established based on updated clinical records. PD patients comparable with MSA only by age, and not disease duration as MSA has a progression faster than PD, were enrolled.

**Table 1:** Demographic and clinical findings of enrolled subjects.

	MSA	PD	HC	*P*
Age, ys (mean ± SD)	62 ± 8	62 ± 8	60 ± 12	0.804
Disease duration, ys (mean ± SD)	3.6 ± 1.3	4.4 ± 2.4	–	0.044
Sex (M/F)	9/11	5/16	5/9	
UPDRS-III (mean ± SD)	–	12 ± 4.15	–	
UMSARS-II (mean ± SD)	41.44 ± 12.88	–	–	

The CECS (Cometico Campania Sud, prot. n°16,544) Institutional Review Board approved this study. Each participant gave written, informed consent for study participation. The study adhered to the tenets of the Declaration of Helsinki.

Patients underwent complete ophthalmic examination, including ChT examination, with an OCT Spectralis (version 6.0.9; Heidelberg Engineering), in sitting and standing positions and IOP measurement, with a Tono-pen Avia (Reichert Technologies) in supine (after 10 min in a quiet room), sitting (after 5 min) and standing position (after 1 min as most MSA patients experience difficulties in longer standing)^19^. In each patient the IOP and the ChT were measured in both eyes, and the results were averaged for the statistical analysis. To avoid fluctuations due to the circadian change in IOP and ChT, the measurements were taken at the same time of the day between 3 and 5. Patients with ocular diseases which may have affected either the IOP or choroidal thickness were excluded from the study. All PD and 10 out of 20 MSA patients were treated with dopaminergic drugs and the ophthalmological examinations were performed on-drug.

Central corneal thickness (CCT) was measured with a Scheimpflug Pentacam HR (Oculus, Wetzlar, Germany, version 1.19r11) to exclude possible CCT effect on the IOP measurements^20^. Moreover, axial length (AL) was measured with an IOLMaster (5.4.4.0006; Carl Zeiss Meditec AG), as a correlation between ChT and AL has been described^21^.

### Statistical analysis

Statistical Analyses were performed with the SPSS package (version 25, SPSS, IBM). Wilcoxon test was used to compare postural changes within the same group. Non-parametric Kruskal–Wallis test was used to compare variations in IOP and ChT after postural changes among the three groups; when significant differences were found among the three groups, a post hoc Mann Whitney U test was used to compare two groups at a time. Spearman correlation analysis was performed to assess relationships between ocular measurements and clinical features in both groups of patients.

## Results

The patients’ demographic and clinical data are summarized in Table 1.

Among the 20 MSA patients, 18 underwent IOP evaluation (one patient refused to have IOP checked and one other was excluded due to a retinal vein occlusion); 9 of them had ChT assessment in both sitting and standing positions ChT was not assessed in 2 patients due to intraocular media opacities and one patient due to a retinal vein occlusion; in the other 9 patients it was not possible to perform the choroidal examination in standing position due to patients difficulties to stand for a long time). Seven out of 18 MSA patients reported no orthostatic symptoms, five reported infrequent orthostatic symptoms that did not restrict activities of daily living (Item 9 UMSARS I score = 1), and six MSA patients reported orthostatic symptoms presenting at least once a week (Item 9 UMSARS I score = 2).

Nineteen PD patients underwent IOP assessment, 18 PD patients had ChT assessment in both sitting and standing positions (in two patients the ChT was not assessed due to intraocular media opacities, whereas another one was excluded due to maculopathy).

No patient was under pharmacological treatment for orthostatic hypotension None of the subjects were affected by systemic diseases potentially affecting IOP such us essential hypertension or diabetes.

Supine to standing IOP variations were significantly higher in MSA vs PD (p = 0.01) and in MSA vs HC (p < 0.0001), whereas no significant differences were observed between PD and HC (p = 0.397) (Tables 2, 3).

**Table 2:** Intraocular pressure (in mmHg) in different positions among the three groups.

	MSA (n = 18)	PD (n = 19)	HC (n = 14)
SUPINE mean ± SD	16.33 ± 2.40	17.2 ± 2.9	16.46 ± 2.66
Range	12–20.5	12–22.5	11.5–20
Sitting mean ± SD	13.28 ± 2.26	14.2 ± 4.1	15 ± 2.94
Range	8.50–17	6 to 21.5	9.5–20.5
Standing mean ± SD	10.85 ± 3.4	13.4 ± 4.9	14.21 ± 2.87
Range	5–17.5	5.5–24.5	8–18.5

**Table 3:** Difference in IOP (in mmHg) between different positions among the three groups.

		MSA	PD	HC
SUPINE-standing	ΔIOP Mean ± SD	5.6 ± 2.82	3.75 ± 3.75	2.25 ± 1.6
	Range	− 0.5 to + 10.5	− 2 to + 13.5	− 1 to + 4.5
	*p	< 0.001	< 0.001	0.002
SUPINE-sitting	ΔIOP mean ± SD	3.03 ± 1.94	3.0 ± 2.81	1.46 ± 1.46
	Range	− 0.5 to + 7	0 to + 11	− 1 to + 4
	*p	< 0.001	< 0.001	0.006
Sitting-Standing	ΔIOP mean ± SD	2.57 ± 1.55	0.75 ± 2.02	0.79 ± 0.85
	Range	0 to + 6.5	− 3 to + 4.5	− 0.5 to + 2.5
	*p	< 0.001	0.117	0.006

*p = Wilcoxon test.

Supine to sitting IOP variations were higher in MSA vs HC (p = 0.014), whereas no significant differences were observed between PD and HC (p = 0.240) and between MSA and PD (p = 0.128).

**Figure 1:**
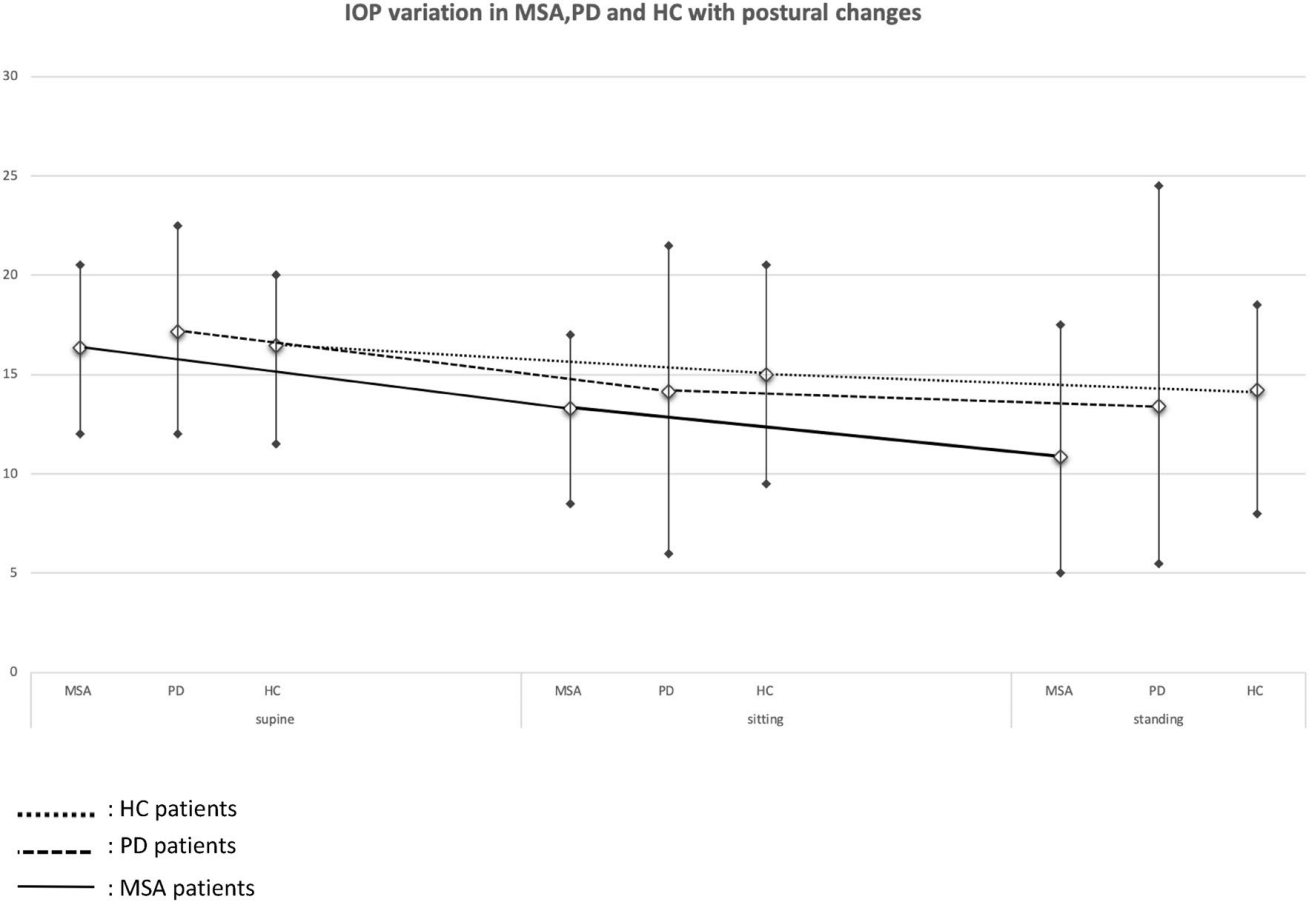
Intraocular Pressure variation (in mmHg) in Multiple System Atrophy, Parkinson’s Disease patients and Healthy Controls with postural changes.

Sitting to standing IOP variations were higher in MSA vs PD (p = 0.005) and in MSA vs HC (p < 0.001), whereas no significant differences were observed between PD and HC (p = 0.760) (Fig. 1).

Sitting to standing ChT variations were significantly higher in MSA vs PD (p = 0.005) and in MSA vs HC (p < 0.001), whereas no significant differences were detected between PD and HC (p = 0.56) (Table 4).

**Table 4:** Choroidal sub-foveal thickness (in µm) among different groups.

	MSA	PD	HC
**Sitting**			
Mean ± SD	240 ± 92	258 ± 79	244 ± 36
Range	101–372	126–399	205–309
**Standing**			
Mean ± SD	215 ± 94	259 ± 76	256 ± 37
Range	82–331	113–382	214–321
**ΔStanding-sitting**			
Mean ± SD	− 25 ± 12	1 ± 28	11.7 ± 6
Range	− 38 to − 4	− 49 to + 54	− 2 to + 20
*p	0.008	0.887	0.007

*p = Wilcoxon test.

MSA patients showed shorter AL than both PD patients (p = 0.003) and HC (p = 0.026) (Table 5).

**Table 5:** Axial length (AL) in mm and central corneal thickness (CCT) in μm among the three groups.

	MSA	PD	HC
AL mean ± SD	23.21 ± 0.95	23.73 ± 0.98	23.65 ± 1.03
Range	22.1–25.59	21.36–25.96	22.05–26.57
CCT mean ± SD	546.25 ± 46.17	548.13 ± 44.40	554.93 ± 31.67
Range	448–635	453–620	500–611

However, the mean sub-foveal ChT in sitting position was not different among groups (p = NS). Mean central corneal thickness (CCT) was not different (p = 0.651) among groups (Table 5).

No relationships were found between ocular measurements and disease severity or duration in MSA and PD patients. No relationships were found between postural changes in both IOP and ChT and Item 9 score of UMSARS I in MSA patients. No differences were found in positional IOP and ChT changes between MSA patients with or without orthostatic hypotension assessed by updated clinical records. No differences were found in IOP and ChT changes between MSA patients on dopaminergic treatment or without it.

## Discussion

The results obtained from this study indicate that MSA patients have significant IOP and ChT postural changes, compared with PD and HC. IOP postural changes have been reported in MSA patients compared with HC; however, to the best of our knowledge, this is the first study to compare MSA with PD patients and to assess postural ChT changes.

A decline in IOP has been described at 1 min from standing in MSA patients compared with HC in two small studies^14,15^, and has been found to correlate with arterial blood pressure decrease due to postural changes^21^. The IOP physiology is complex and incompletely understood. IOP is mainly determined by the rates of aqueous humour production and drainage^22,23^. Arterial and venous pressures changes may have transient effects on IOP^24^. Raising in venous pressure can reduce aqueous drainage and lead to IOP increase^25,26^. However, as aqueous drainage is relatively slow^27–29^, it is unlikely that arterial blood pressure changes may account for the rapid IOP decline observed in our study, and it is possible that orthostatic hypotension and IOP decrease independently reflect the autonomic dysfunction of MSA.

The rapid IOP changes observed in our study are probably due to direct pressure and volume changes in the vascular compartments within and around the eye, including the periorbital tissues and the intraocular blood volume, most of which lies in the choroid^30^.

Indeed, our finding of a significant ChT reduction in MSA patients compared with PD and HC in the standing position, paralleling IOP decrease, supports the hypothesis that choroidal blood volume regulates the production of aqueous humor and ocular tension.

In 1992 Philips et al.^24^ introduced the concept that blood pressure plays a role in the regulation of ocular tension (the aqueous humor in the eye). They found a correlation between the pulsation of the eye and ocular tension, suggesting that the choroidal blood flow, influenced by the blood pressure, could work like a “piston”, regulating the production and the outflow of aqueous humor.

Changes in IOP according to the body position have been described^31–35^. In a recent study the IOP increase, observed in HC passing from sitting to upside-down position, was related to choroidal thickening^36^.

To the best of our knowledge, this is the first study to analyze the postural changes-related IOP behavior in PD patients. However, we found that IOP changes in PD patients were similar to HC subjects, whereas positional changes in IOP were significantly higher in MSA patients vs both PD patients and HC.

A study that analyzed the effect of posture on blood and IOP in patients with autonomic dysfunction was conducted by Singleton et al.^14^. They found a correlation between mean arterial pressure postural changes and IOP postural changes, confirming the importance of the baroreflex system in this mechanism. They did not analyze the ChT, but they used the value of mean ocular perfusion pressure (MOPP), defined as mean arterial pressure (MAP) minus IOP.

In our study, we found a greater reduction in ChT from sitting to standing position in MSA patients compared with both PD and HC. A more preserved baroreflex system in patients with PD compared to MSA could explain the lack of significant differences between PD and HC.

An inverse relationship between AL and ChT has been described in the literature^21^, however in this study ChT evaluation in sitting position showed a non-significant difference among the three groups, even though MSA patients showed shorter AL than both PD patients and HC. These results suggest that MSA may have thinner ChT compared to the other groups, despite the shorter AL, but further studies in a larger population relating AL ranges with ChT are needed to confirm this finding.

In conclusion, our results show that patients with MSA, more than PD, may have IOP fluctuations during the day, depending on postural changes; this factor should be considered in their ophthalmic management when a treatment for ocular hypertension is planned. IOP changes, seemingly, did not parallel orthostatic symptoms or orthostatic hypotension in MSA patients, suggesting that the altered postural IOP changes may develop independently from orthostatic hypotension. However, we must recognize that supine and orthostatic blood pressure was not assessed at the same time of ophthalmic examination for compliance reasons, thus possibly affecting such findings.

Furthermore, in this study we first demonstrate a reduction in ChT from sitting to standing position in MSA patients, which could be considered as an additional hallmark of autonomic dysfunction in this disease. Additional studies in MSA patients and HC are needed to better understand the effects of IOP changes on ocular and choroidal hemodynamics. Moreover, we suggest that IOP and ChT changes may deserve further studies to prove their feasibility as possible biomarkers to differentiate MSA and PD in early disease stages.
